# Gonadal mosaicism and non‐invasive prenatal diagnosis for ‘reassurance’ in sporadic paternal age effect (PAE) disorders

**DOI:** 10.1002/pd.5108

**Published:** 2017-08-01

**Authors:** Andrew O. M. Wilkie, Anne Goriely

**Affiliations:** ^1^ Clinical Genetics Group, Weatherall Institute of Molecular Medicine University of Oxford Oxford UK

Non‐invasive prenatal diagnosis (NIPD) holds great promise to increase the options for women seeking prenatal testing, as it combines the benefits of earlier sampling in pregnancy with absence of procedure‐related risk to the fetus. But all new procedures have costs and, in an article published in *Prenatal Diagnosis* last year, Verhoef *et al*.^1^ undertook a health economic evaluation of NIPD in the UK. They noted that ‘In [a] cash‐constrained publicly funded health care system such as the English NHS, decisions about the broad programme of NIPD tests are likely to be largely dependent upon their relative costs’ and advocated that ‘careful audit of any NIPD service should be put in place from the outset’. Here, we wish to highlight that in prenatal testing for the (remote) possibility of sibling recurrence of so‐called paternal age effect (PAE) disorders (such as achondroplasia, thanatophoric dysplasia and Apert syndrome), such NIPD (estimated cost of £550 per procedure) is *already* taking place (including in the authors' own institution), without the health economic case having been clearly assessed. We believe that if such an assessment was undertaken, this particular use of NIPD may be difficult to justify at current prices.

To explain and clarify our point of view, we note that there are three different contexts in which NIPD may take place because an increased risk of a dominant genetic disorder is suspected in the fetus^2^: (1) one of the parents is themselves affected (risk 50%); (2) fetal abnormality is detected (risk depends on positive predictive value of test/findings); and (3) a sibling of the fetus has an apparently sporadic dominant mutation (risk is related to the possibility of gonadal mosaicism in one of the parents). Our concerns relate to this third scenario: Whilst studies of gonadal mosaicism suggest an overall risk of 1–2% for point mutations^3^ and up to 4% for chromosomal rearrangements^4^ (cost £13 750–£55 000 per case detected, economically justified), in the specific context of PAE disorders, the risk is likely to be at least an order of magnitude lower, so the economic argument is much more finely balanced.

What do we mean by a ‘PAE disorder’? We previously highlighted^5^ that a small number of dominant genetic conditions, most commonly caused by mutations in the genes *FGFR2*, *FGFR3*, *HRAS*, *KRAS*, *PTPN11*, and *RET*, show a striking combination of epidemiological and molecular features that distinguish them from the bulk of disease‐causing mutations. Briefly summarised, these features comprise (1) a very high apparent germ line mutation rate, (2) extreme bias towards a paternal origin of mutations, (3) markedly elevated age of the healthy father (this is the PAE by which these disorders are collectively known) and (4) the causative mutations confer gain‐of‐function to the encoded proteins, which are involved in signalling through the growth factor receptor‐RAS‐mitogen‐activated protein kinase (MAPK) pathway. An alternative collective term is ‘RAMP’ (recurrent, autosomal dominant, male‐biased, PAE).^6^ We have demonstrated that PAE disorders arise in testes by clonal expansion of rare mutations along segments of seminiferous tubules, through a mechanism termed selfish spermatogonial selection.^7^, ^8^ This process occurs during adulthood; hence, despite positive selection, the mutations never attain the levels in sperm associated with classical gonadal mosaicism, which arises during early embryonic development. For example, in a total of 909 published measurements in sperm of four different PAE mutations causing Apert syndrome,^9^, ^10^ thanatophoric dysplasia^11^ or Costello syndrome,^12^ the highest specific mutation level measured in any sample was 1 in 1380.^10^

In the context of NIPD, the unique characteristics of PAE disorders make them particularly suited to its use and (potentially) misuse. Indeed, stimulated by the knowledge that PAE mutations are the most common *de novo* germ line mutations in the human genome, have a narrow mutation spectrum and originate nearly exclusively from the healthy father, specific assays for several of these disorders were amongst the first to be reported (for achondroplasia/*FGFR3*,^2^, ^13^, ^14^ thanatophoric dysplasia/*FGFR3* 2, 14 and Apert syndrome/*FGFR2*).^2^ Based on scrutiny of the indications for NIPD presented in these reports^2^, ^14^ as well as in the article by Verhoef *et al.*,^1^ it is apparent that in many instances, NIPD has been performed to detect recurrence of a *de novo* mutation (indication 3 earlier), rather than because of 50% prior risk or fetal abnormality.

**Figure 1:**
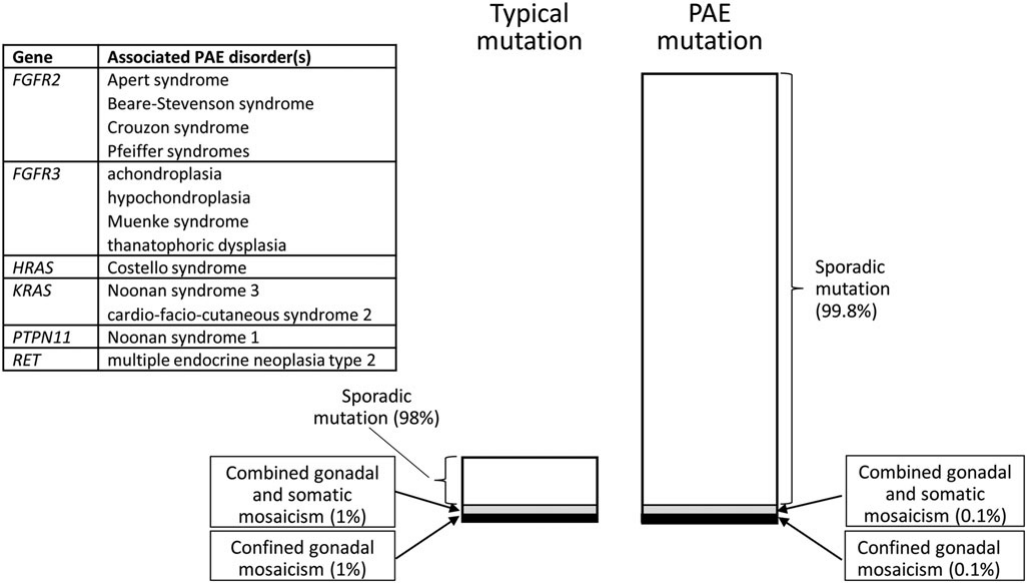
Diagrammatic representation of the effect of paternal age effect (PAE) mutations on the proportion of cases of gonadal mosaicism. Number of mutations is proportional to area of boxes. Although mosaicism (grey and black boxes) may still occur at the background frequency, its relative prevalence is substantially diluted out for PAE compared with typical mutations, because selfish spermatogonial selection strongly enriches for sporadic mutations (unfilled boxes) originating from the father. In the example shown, ~90% of mutations are attributable to the PAE, but for the most abundant mutations (such as those causing achondroplasia, thanatophoric dysplasia or Apert syndrome), the effect may be more than an order of magnitude greater. The table insert lists the most common congenital disorders caused by PAE mutations.

What is the evidence base to justify NIPD for sibling recurrence in PAE disorders owing to the risk of parental confined gonadal mosaicism in one of the parents? We do not argue that this cannot occur; we are aware of eight reports of proven gonadal mosaicism for a recognised PAE mutation,^15^, ^16^, ^17^, ^18^, ^19^, ^20^, ^21^, ^22^ although in only four of those^16^, ^18^, ^21^, ^22^ was the mutation occult (that is, not also detectable in a parental blood sample). Rather, rare mosaic cases become swamped out in PAE disorders by the much more frequent process of adult male‐driven PAE mutation, so the relative risk of high‐level mosaicism is substantially lower than for usual, non‐PAE mutations (Figure 1). It is difficult to accurately determine the relative risk, because for the rare reported mosaic events, the denominator (total *de novo* mutations from which those events were sampled) is unknown. The only large empiric study that we are aware of, in achondroplasia, found a single recurrence (not molecularly proven) in 443 siblings (~0.2%).^23^ A simple calculation based on the known biology of PAE disorders would suggest that the risk of mosaicism is inversely proportional to the elevation in mutation rate owing to selfish selection. For common PAE disorders, the multiplier for the mutation rate ranges from 10 to >100, so that the relative risk of mosaicism would be one tenth to one hundredth the usual risk, or 0.2–0.02%. Put another way, using the number needed to treat (NNT), a common metric in drug trials and health economics, the NNT for NIPD applied to detection of sibling recurrence of common PAE mutations is likely to range from the hundreds to the thousands. At these extremely low risk levels, the apparent benefits of a negative NIPD for maternal ‘reassurance’ and avoidance of additional sonographic screening later in the pregnancy^2^ are actually rather misleading, because this may divert attention from the substantially higher 2–3% baseline risk of serious congenital or genetic disorder faced in all pregnancies, and for which normal screening recommendations should still apply.

In conclusion, we urge that clinical geneticists, genetic counsellors, obstetricians and midwives should have greater awareness of the distinct biological features of PAE mutations (Figure 1) when counselling about the sibling recurrence risk after the birth of a child with one of these *de novo* mutations and (after confirming the absence of the mutation in parental blood samples) reassure the couple that the recurrence risk is extremely low. Given that the risk of causing the miscarriage of a healthy fetus likely exceeds the risk of the PAE disorder being tested for, we believe that invasive prenatal diagnosis to look for sibling recurrence of a PAE mutation is difficult to justify on medical, ethical or economic grounds. Whether NIPD is justified in the same context is not clear and should be the subject of further debate and research. What is clear is that the prospective collection of NIPD data should distinguish this from other indications for testing, which would provide the opportunity to measure the NNT in an empirical setting.

Looking to the future, we previously highlighted^5^ that PAE mutations have characteristics that could make them ideally suited to implementing non‐invasive screening (as opposed to targeted testing) in harness with screening for Down syndrome.^24^ Recently, a commercially available screening test (PreSeek™) has appeared on the market that apparently includes several PAE mutations; as yet we are unaware of published data supporting the sensitivity and specificity of this new application.
